# Application of internet-based cognitive behavioral therapy for negative emotions in pregnant women: a narrative review

**DOI:** 10.3389/fpsyt.2026.1896124

**Published:** 2026-08-05

**Authors:** Zhiliu Wan, Yaping Liu, Zhengli Su, Caiying Bai, Xiaoling Liu, Xuejiao Li, Xu Zhao, Pan Li, Li Song, Jie Rao

**Affiliations:** 1 School of Nursing, Gansu University of Chinese Medicine, Lanzhou, Gansu, China; 2 Department of Medicine, Northwest Minzu University, Lanzhou, Gansu, China; 3 Department of Obstetrics, Gansu Province Maternity and Child Health Hospital (Gansu Provincial Central Hospital), Lanzhou, Gansu, China; 4 Department of Pediatric Emergency, Gansu Province Maternity and Child Health Hospital (Gansu Provincial Central Hospital), Lanzhou, Gansu, China; 5 School of Medical Information Engineering, Gansu University of Chinese Medicine, Lanzhou, Gansu, China

**Keywords:** ICBT, internet-based cognitive behavioral therapy, negative emotions, pregnant women, review

## Abstract

During the gestational period, pregnant women commonly experience negative emotional states including anxiety, depression, and childbirth fear, which are induced by dramatic physiological and psychological changes. These negative emotional disturbances not only damage maternal physical and mental health, but also may increase the risk of adverse fetal outcomes and long-term developmental abnormalities in offspring. Internet-based cognitive behavioral therapy (ICBT) is a psychological intervention modality developed on the basis of cognitive behavioral theory and delivered through network platforms. In recent years, ICBT has received extensive attention in the intervention of negative emotions among pregnant women. Its main advantages include breaking through temporal and spatial constraints, reducing intervention costs, and improving the accessibility of psychological services. Therefore, this paper systematically reviews the conceptual connotation and core components of ICBT, and analyzes its application effects in the intervention of anxiety, depression, and childbirth fear among pregnant women, aiming to provide a reference for subsequent related research and clinical practice of ICBT in this population.

## 1 Introduction

Pregnancy is a critical developmental stage marked by dramatic physiological and psychological changes. In this period, pregnant women often experience significant stress responses, which further induce negative emotional states including anxiety, depression and fear of childbirth. Existing epidemiological evidence indicates that the prevalence of anxiety symptoms among pregnant women reaches up to 40%, while the prevalence of depressive symptoms reaches up to 25% (1); approximately 14% to 22% of pregnant women experience fear of childbirth, among which 6% to 10% suffer from severe fear of childbirth (2). Gestational negative emotions not only increase the incidence of cesarean section and postpartum mental disorders, but are also significantly associated with adverse outcomes such as preterm birth, low birth weight and long-term developmental abnormalities in offspring (1). Therefore, the implementation of timely and effective interventions for maternal mental health is of great clinical significance for protecting the physical and mental health of both mother and child.

Interventions for gestational negative emotions are mainly categorized into pharmacological and non-pharmacological approaches. Non-pharmacological interventions can avoid potential adverse effects of psychotropic medications on maternal and infant health, and are therefore widely adopted in the clinical management of negative emotions among pregnant women. Common interventions in this category include cognitive behavioral therapy (CBT), mindfulness-based therapy, acceptance and commitment therapy (ACT), interpersonal psychotherapy (IPT), psychoeducation, and mind-body exercises, among others. Among the above interventions, CBT is recognized as a widely applied and effective non-pharmacological intervention for gestational negative emotions (3, 4), and can be used to regulate dysregulated emotions, behaviors and cognitions (5). However, traditional face-to-face CBT has certain limitations, including high treatment costs, difficulties in implementation, and restrictions on time and geographic access (5). This bottleneck is particularly prominent in low- and middle-income countries with a general shortage of professional mental health practitioners (6). In contrast, internet-based cognitive behavioral therapy (ICBT) breaks through the constraints of cost, time, space and the availability of therapists (7), conforms to the development demand of the current digital medical era, and has become a feasible alternative to traditional face-to-face CBT (8, 9). Mindfulness-based therapy is a kind of meditative intervention that can effectively relieve prenatal emotional disorders, emotional distress and psychological stress. However, existing evidence has not confirmed that its efficacy is superior to other types of interventions, and it is not suitable for all pregnant women, especially those with severe depressive symptoms before delivery (10, 11). As a third-wave cognitive behavioral therapy, ACT adopts mindfulness and acceptance strategies to promote targeted behavioral change (12). Its core advantage is that it is suitable for online self-help implementation, and has unique transdiagnostic application potential, which may improve long-term mental health outcomes beyond the perinatal period (13). IPT is a time-limited psychotherapy that focuses on the relationship between individual mental state and interpersonal interaction, and is especially helpful for perinatal women to adapt to psychosocial role transformation and cope with interpersonal conflicts. However, this intervention puts forward high requirements for therapists, who need to have solid interpersonal intervention concepts and professional therapeutic skills (14, 15). Psychoeducation provides targeted health information and professional knowledge to guide pregnant women to develop healthy emotional and behavioral patterns. Although this intervention is low-cost and the most accessible, differences in health literacy among pregnant women may affect individual intervention adherence (16). Mind-body exercises improve individuals’ self-regulation ability by regulating the interaction among brain, mind, body and behavior. However, this intervention is not recommended for pregnant women with moderate to severe negative emotional symptoms, and there may be potential safety risks in implementation (17). At present, the application of ICBT in relieving negative emotions of pregnant women has been relatively well developed globally, but it is still in the initial exploratory stage in China. Therefore, this paper systematically reviews the concept, core components and application progress of ICBT for gestational negative emotions, aiming to provide a theoretical basis for the subsequent development of maternal mental health services in China.

This article is a narrative review. Rather than conducting a systematic quantitative synthesis, this review aims to provide a comprehensive and critical overview of the current state of evidence regarding the application of ICBT for negative emotions in pregnant women. The scope of this review was clearly defined according to the following dimensions: (1) population (P): Pregnant women across all trimesters, including perinatal populations, with or without clinically diagnosed mental health conditions. (2) Concept (C): ICBT interventions delivered via digital platforms (e.g., websites, mobile applications, videoconferencing software, social media tools), including both self-guided and therapist-assisted formats. (3) Context (C): Studies conducted in any geographic setting (both high-income and low- to middle-income countries), reported in English or Chinese, and published in peer-reviewed journals. (4) Outcomes of interest: Negative emotional states, including but not limited to anxiety, depression, and fear of childbirth. The search terms in both Chinese and English covered terminology related to “internet-based cognitive behavioral therapy, ICBT, cognitive behavioral therapy, cognitive therapy, behavioral therapy, cognitive behavioral treatment, Internet, remote, mobile health, online, telenursing, telecommunications, computer, mobile, network, cognitive psychotherapy, CCBT, pregnant women, maternal, parturient women, normal pregnant women, pregnant women, perinatal period.” Given the narrative nature of this review, no standardized quality assessment instruments (such as the Cochrane Risk of Bias Tool or the Newcastle-Ottawa Scale) were used. This review primarily focuses on three aspects: (1) an overview of ICBT; (2) core components of ICBT (including platforms, frequency and duration, and intervention content); and (3) the application of ICBT to three specific negative emotions in pregnant women-anxiety, depression, and fear of childbirth.

## 2 Overview of internet-based cognitive behavioral therapy

CBT is fundamentally an integration of two distinct psychotherapeutic approaches: cognitive therapy (CT) and behavior therapy (BT). BT emerged first, originating in the 1950s and 1960s from the application of behaviorist principles to facilitate behavioral change in humans (18). The philosophical roots of CT can be traced back to the Stoic philosophers of ancient Greece, and it began to develop in the United States in the mid-1960s. Its founder, Aaron Beck, is widely regarded as the most influential figure in CBT. In the 1980s, Beck integrated BT into CT, formally establishing CBT as a comprehensive therapeutic framework. Its core components include behavioral activation, exposure exercises, behavioral experiments, relaxation training, and social skills training (5, 18, 19). In the 1990s, the third wave of CBT emerged, encompassing approaches such as Mindfulness-Based Stress Reduction (MBSR) and Acceptance and Commitment Therapy (ACT) (18). Subsequently, CBT gained widespread adoption across the United Kingdom, North America, Australia, and parts of Europe (19).

Since the 1990s, the rapid proliferation of internet technology has provided a technological impetus for innovations in mental health service delivery. Researchers began exploring the integration of core CBT principles with internet platforms, aiming to overcome the temporal and spatial constraints of traditional therapy while reducing implementation costs and expanding service accessibility. In 1999, a Swedish research team conducted the world’s first clinical trial of internet-based cognitive behavioral therapy (ICBT) (20). Vernmark et al. (21) further noted that Sweden has consistently remained at the forefront of ICBT development, both in research and routine clinical practice. Around the same period, research teams in the United States and the Netherlands also initiated ICBT investigations. Currently, ICBT research has evolved into a global academic field, attracting researchers worldwide who collectively advance its ongoing development. This international collaboration was exemplified by the 12th Scientific Meeting of the International Society for Research on Internet Interventions (ISRII), held in Ireland in 2024, which brought together nearly 450 delegates from 27 countries to present the latest scientific findings. High-impact research teams in Australia, Germany, the Netherlands, Denmark, Norway, the United Kingdom, Spain, Switzerland, Canada, New Zealand, and the United States have made significant contributions to the theoretical refinement and clinical translation of ICBT (21, 22).

ICBT is an emerging psychological intervention modality developed on the theoretical basis of cognitive behavioral therapy. This intervention is delivered via internet platforms, and enables patients to identify, assess and adjust emotional distress and maladaptive behaviors caused by cognitive dysfunction (23). By utilizing electronic devices including computers and mobile terminals, ICBT adopts visual and multi-media presentation forms such as images and videos to correct patients’ cognitive bias toward their own conditions, so as to improve individual problem-solving ability (24). Currently, ICBT has not only been widely used in the intervention of general mental disorders (25), but also expanded to psychological intervention for special populations and somatic diseases, covering gestational diabetes mellitus (5, 9), pregnancy termination for fetal abnormalities (26), perinatal insomnia (27), irritable bowel syndrome (28) and cancer (29), etc.

ICBT can be primarily divided into two categories: self-guided therapy and therapist-assisted therapy (25). Compared with therapist-guided ICBT, self-guided ICBT has a wider coverage, but its patient engagement and clinical benefits are relatively lower (30). This model emphasizes the autonomy of patients, allowing them to independently learn the core theories and techniques of CBT through pre-designed online courses and resources, with minimal involvement from therapists. In contrast, therapist-assisted therapy integrates regular therapist guidance on the basis of the self-guided learning framework. Therapists provide timely feedback and dynamic adjustment for patients’ learning process through emails, online messages or short video calls. This not only breaks through the time and space limitations of traditional in-person psychotherapy, but also provides professional support at key nodes, which effectively promotes the transformation of theoretical knowledge into practical emotion regulation strategies (31). Nevertheless, the two intervention modes differ in efficacy, treatment adherence and cost-effectiveness. First, a large amount of existing evidence shows that therapist-assisted ICBT has higher efficacy than self-guided ICBT in the intervention of various mental disorders (30, 32). For example, based on intention-to-treat analysis, March et al. (30) found that at the 9-month follow-up, 69% of participants in the therapist-assisted group achieved negative conversion of the primary anxiety diagnosis, while this proportion was only 41% in the stepped-care group (initial self-guided treatment, with additional therapist assistance for non-responders). Further stratified analysis showed that therapist-assisted therapy brought more significant clinical benefits for patients with moderate to severe symptoms, while patients with mild symptoms can obtain equivalent positive outcomes without real-time therapist guidance. In other words, the advantage of therapist-assisted therapy may be more prominent in patients with moderate to severe symptoms (25, 33). The above conclusions are supported by another three-arm randomized controlled trial (32), which further confirmed that therapist-guided ICBT has higher efficacy than unguided self-guided ICBT. However, a meta-analysis conducted by Oey et al. (34) reached different conclusions: the overall effect sizes of therapist-guided and self-guided ICBT are comparable, and only the short-term efficacy of the former has a weak edge. Multicenter large-sample clinical studies are still needed to further clarify the difference in efficacy between the two modes. Second, the dropout rate of self-guided therapy is generally higher than that of therapist-assisted therapy (25). March et al. (30) found that the average number of completed treatment sessions in the self-guided group was (7.39 ± 3.44), which was lower than that in the therapist-assisted group (8.73 ± 3.08). This result is consistent with the conclusion of Richards et al. (35), who pointed out that therapist participation can reduce the dropout rate by 30% to 40%.

In terms of cost-effectiveness, Lundström et al. (36) carried out a comprehensive analysis from three dimensions: therapist working time, direct medical cost and indirect cost. The study showed that the average weekly working time of therapists in the face-to-face CBT group was 120.4 minutes (95% CI: 115.4-125.3), while that in the therapist-guided ICBT group was 10.6 minutes (95% CI: 8.9-12.4); the unguided ICBT group only required 60 minutes of fixed administrative work throughout the treatment period. In terms of direct treatment cost, the average cost of face-to-face CBT was about $6795 (SD 237), while that of therapist-guided ICBT was $603 (SD 176), and that of unguided ICBT was $249 (SD 168). Compared with face-to-face CBT, both types of ICBT have better cost-effectiveness, and the direct treatment cost of unguided ICBT is lower than that of therapist-guided ICBT. However, in terms of indirect cost, compared with face-to-face CBT, therapist-guided ICBT is expected to save $6153 (95% CI: 4536-7563, *P* < 0.001), while unguided ICBT is expected to save $5761 (95% CI: 4145-7298, P < 0.001). Notably, the indirect cost saving of therapist-guided ICBT is slightly higher than that of unguided ICBT. On the whole, among the three modes, self-guided ICBT has the lowest direct treatment cost, but its indirect cost saving effect is not as good as that of therapist-assisted ICBT. In conclusion, the two intervention modes of ICBT are complementary to each other, and jointly form a flexible and efficient implementation mode of ICBT, which can provide diversified intervention options for patients with different needs and different situations (23).

## 3 Core components of ICBT

### 3.1 Intervention platforms

Current ICBT interventions targeting pregnant women primarily adopt web- and application-based implementations, covering platforms including WeChat, Tencent Meeting (37), DingTalk, video channels (38), the Motherly application (6), Google Meet, e-mail (39), the Luna Luna Baby application (40, 41), the PandaMom program (42), the Sunnyside shared website (43), video calling software (44), the manualized UPLIFT program (45), general software programs (46), and the THP-Brief Group Version (THP-BGV) (47). While the effectiveness of these platforms in alleviating negative emotions among pregnant populations has been empirically validated, existing platforms exhibit substantial heterogeneity in functional design, interactivity, and user engagement. For instance, the Luna Luna Baby application provides modular intervention content with a short single-session duration, which is adaptable to the fragmented daily schedule of pregnant women (48). By contrast, videoconferencing-based platforms, despite their favorable interactivity, impose higher time requirements on users, which may negatively correlate with intervention adherence (49). It is worth noting that platform selection not only affects user experience, but may also exert a direct impact on final intervention outcomes. Future studies are suggested to conduct comparative analysis on the effectiveness of different intervention platforms, and leverage artificial intelligence and machine learning techniques to achieve personalized internet-based interventions (31), and to realize remission prediction for major depressive disorder patients based on multimodal data (50).

### 3.2 Intervention frequency and duration

At present, there is no standardized optimal intervention frequency or duration for ICBT targeting pregnant women, and considerable heterogeneity exists across existing studies, which reflects the exploratory developmental stage of this research field. For example, Haga et al. (51) designed an intervention program consisting of 44 10-minute sessions, with 11 sessions arranged during pregnancy and 33 sessions arranged in the postpartum period. Although each single session is short in duration, the overall high intervention frequency may bring additional challenges to pregnant women who already bear physical and psychological pressure, which may ultimately lead to low intervention adherence. In contrast, Shariatpanahi et al. (8) adopted an intervention protocol containing 8 sessions, each with a duration of 50 minutes. Even though the total number of sessions is reduced, the longer duration of individual sessions also increases the risk of participant dropout caused by fatigue or time conflicts. Puertas-Gonzalez et al. (39) examined a high-intensity intervention protocol, which included 8 weekly sessions with a duration of 1.5–2 hours per session. Such a high-density intervention arrangement may increase the physical and psychological burden of pregnant women and further reduce intervention adherence.

Existing research evidence indicates that there is a clear correlation between intervention intensity and intervention adherence among pregnant women. A meta-analysis focused on participants with mild depression (52) showed that 6 sessions is the most frequent intervention arrangement adopted in existing studies, implying that moderately low-frequency interventions may have higher acceptability among pregnant women. However, there is still a lack of randomized controlled trials that directly compare the intervention effects of different frequencies and durations on improving negative emotions in pregnant women. Future research needs to develop phased and differentiated intervention protocols based on the physiological and psychological characteristics of different trimesters of pregnancy, balance intervention efficacy and participant burden reduction, so as to improve intervention adherence and provide a more reliable empirical basis for the standardization of ICBT protocols.

### 3.3 Intervention content

Numerous studies (39, 53, 54) have demonstrated that the content of ICBT mainly covers psychoeducation, mood monitoring and training, cognitive restructuring, behavioral activation, relaxation training, social skill training, problem-solving and relapse prevention strategies. Intervention content is delivered through multiple media formats, including images, text, audio, video, animation and tutorials (31). Current studies show significant heterogeneity in the emphasis of intervention modules. For instance, Takae et al. (40) and Nishi et al. (41) both adopted the “Luna Luna Baby” application to deliver six ICBT modules, namely psychoeducation, case formulation based on cognitive-behavioral model, behavioral activation, self-compassion, mindfulness and problem-solving. On the basis of the standard ICBT protocol, Duan et al. (55) supplemented four modules covering anxiety and worry, sleep problems, interpersonal relationship and the development of maternal-infant relationship, which has been proven to exert positive effects on mild-to-moderate antenatal depressive symptoms and the prevention of postpartum depressive symptoms. Shahsavan et al. (46) implemented an 8-week ICBT intervention that only focused on four modules: self-monitoring, cognitive restructuring, relaxation and confidence building, and problem-solving, and obtained positive intervention outcomes. In contrast, another study (56) adopted an intervention program consisting of eight modules covering mental health, sleep, diet, physical activity and other topics, which was combined with online brief cognitive behavioral therapy (b-CBT). However, compared with the control group that received an educational application plus online b-CBT, this intervention did not show a significant effect. A potential explanation is that b-CBT itself has produced a considerable therapeutic effect, leaving limited marginal space for improvement after adding extra intervention components. In addition, participants may face operational difficulties when using the application or fail to achieve sufficient intervention engagement. Furthermore, the number of prenatal visits was found to be correlated with randomization status; higher frequency of prenatal care may have a positive impact on the outcome, thus introducing confounding bias. Although the between-group comparison failed to confirm the additional benefit of the application, about 80% of participants showed improvement in depressive symptoms after receiving the intervention, with a large within-group effect size. The above findings indicate that the online b-CBT program, whether or not supported by smartphone applications, may be a promising intervention for maternal depression in low- and middle-income countries (LMICs).

It should be emphasized that all the aforementioned ICBT intervention modules are not independently developed, but are directly derived from the traditional CBT system that has been verified by decades of empirical research. Cognitive restructuring is theoretically based on Beck’s cognitive theory (18), while behavioral activation, which is rooted in behavioral learning theory, has been confirmed to be effective in improving blood glucose control and enhancing self-efficacy in patients with gestational diabetes mellitus (57). Relaxation training (such as diaphragmatic breathing and progressive muscle relaxation) functions through the “relaxation response” mechanism in psychophysiology, and can effectively reduce the level of stress and anxiety during pregnancy (39, 58). The theory and practice of mindfulness can be traced back to Buddhism and other spiritual traditions thousands of years ago. The third-wave cognitive behavioral therapy, which emerged in the 1990s, integrated concepts such as acceptance, mindfulness and non-judgmental awareness into the traditional CBT framework. Up to now, the efficacy of mindfulness-based interventions in improving negative emotions of pregnant women has been fully verified (10, 59). The concept of self-compassion is highly consistent with the “Self-in-Relation Model” of female psychological development proposed by Judith Jordan. At the same time, it also responds to the viewpoints of multiple humanistic psychologists, including Rogers’ “unconditional positive regard”, Ellis’ “unconditional self-acceptance” and Maslow’s emphasis on self-acceptance and self-knowledge (60). In recent years, as a core component of the third-wave CBT, self-compassion has been integrated into intervention frameworks such as Acceptance and Commitment Therapy (ACT), Compassion-Focused Therapy (CFT) and Mindfulness-Based Cognitive Therapy (MBCT). These interventions have been delivered as standardized modules to special groups such as pregnant women through network platforms (such as the Luna Luna Baby application) for the management of negative emotional states (40, 41).

To sum up, the essence of ICBT does not lie in the innovation of therapeutic technology, but in delivering the empirically supported core components of CBT in a structured and standardized manner through digital platforms, so as to improve the efficiency of psychological intervention and alleviate the negative emotional symptoms of pregnant women. For details, see **Table 1**.

**Table 1:** Summary of internet-based cognitive behavioral therapy interventions.

Study	Platforms	Frequencies	Durations	Intervention content	Outcomes
Cao et al. (37)	WeChat and Tencent Meeting	1 module per week, with an additional one-on-one online session in Week 6.	7 weeks	knowledge and skill learning, online group discussion.	improved depression, anxiety, and fear of childbirth.
Wu et al. (38)	DingTalk	cognitive intervention: 6 video sessions, once weekly, 40–60 min each; behavioral intervention: 3 scenario-based simulation sessions, once weekly on Saturday evenings, 60 min each.	9 weeks	cognitive modules (6 video sessions): “Perinatal Mental Health and Emotional Management”, “How to Relieve Labor Pain”, “Benefits of Natural Delivery”, “Cooperation in Delivery”, “Inside the Delivery Room”, and “Case Sharing of Safe Vaginal Delivery After Cesarean Section”.	the intervention significantly alleviated childbirth-related fear and anxiety, and notably improved the rate of vaginal delivery.
Zuccolo et al. (6)	Motherly App	4 brief CBT sessions completed within 8 weeks.	8 weeks	(1) mental health; (2) sleep; (3) nutrition; (4) physical activity; (5) social support; (6) prenatal support; (7) postnatal support, (8) library of pre- and postnatal content.	–
Puertas-Gonzalez et al. (39)	Google Meet and email	one weekly group session, 1.5–2 h each.	8 weeks	(1) psychoeducation; (2) deactivation techniques (diaphragmatic breathing and thematic imagination); (3) cognitive restructuring: cognitive distortions; (4) cognitive restructuring: irrational beliefs; (5) other complementary strategies: self-instructional training and time organization; (6) training in social skills: assertiveness, basic assertive rights, saying no and how to request a change of behaviour; (7) relationship between anger and stress and emotional self-regulation; (8) good mood and optimism: summary.	lower perinatal stress and anxiety, higher psychological resilience, and reduced depressive and obsessive-compulsive symptoms.
Nishi et al. (41) and Takae et al. (40)	Luna Luna Baby App	6 modules, one module per week, 5 min each.	from 16–20 weeks of gestation to 32 weeks (third trimester)	(1) psychoeducation; (2) case formulation based on a cognitive-behavioral model; (3) behavioral activation; (4) self-compassion; (5) mindfulness; (6) problem-solving.	preventive effects against perinatal depression in pregnant women with mild depressive symptoms.
Schmidt-Hantke et al. (42)	PandaMom	a 10-module program, with users advised to complete one module per week according to their individual needs.	outcome assessments were conducted at baseline (pre-intervention), and at 2 weeks and 5 weeks postpartum.	(1) audio-visual content; (2) psychoeducational materials; (3) exercises, and self-report questionnaires.	the program demonstrated satisfactory feasibility and acceptability among the target population.
Duffecy et al. (43)	Sunnyside Share website	16 sessions delivered twice weekly, 10–15 min per session.	8 weeks	(1) your mood and your pregnancy; (2) worries about me and my Baby; (3) mood management; (4) challenging your thinking; (5) positive activity during pregnancy; (6) physical activity during pregnancy; (7) partner communication and support; (8) body image and sex during pregnancy and the postpartum; (9) relationships with your mother and mother-in-law; (10) challenges in relationships with friends and others; (11) monitoring kick counts and other pregnancy anxiety; (12) anxiety and parenthood; (13) relaxation; (14) employment issues; (15) during and after the birth: how to manage and resources; (16) moving forward and conclusions.	significant improvement in postpartum depressive symptoms was observed.
Okatsu et al. (44)	Video call software	three CBT sessions, 30 minutes per session.	intervention delivered from the prenatal period through 1 month postpartum.	psychoeducation and cognitive behavioral therapy components.	significant improvement in anxiety symptoms was observed.
Latendresse et al. (45)	UPLIFT manualized program	one 60-minute session per week.	8 weeks	(1) check-in; (2) discussion; (3) teaching, skills building; (4) a group exercise, and “on your own” assignments.	the program was found to be feasible for implementation in pandemic-affected environments, resource-constrained settings (e.g., rural areas), and underserved minority communities.
Shahsavan et al. (46)	Software-based program	–	delivered throughout the entire pregnancy.	(1) self‐monitoring; (2) cognitive restructuring; (3) relaxation; (4) assertiveness; (5) problem‐solving.	the intervention significantly improved fear of childbirth, anxiety, depression, and stress, and reduced the proportion of women with a preference for cesarean section.
Boran et al. (47)	THP-BGV program	five online group sessions delivered once weekly, 60 minutes per session.	5 weeks	(1) engagement and introduction to the program; (2) psychoeducation and problem-management skills; (3) focusing on one’s personal health and well-being; (4) establishing the mother-infant bond; (5) reactivating relationships with others and closing the therapy.	the intervention was associated with mild reductions in anxiety and depressive symptoms.
Haga et al. (51)	Mamma Mia	–	11.5 months	the intervention comprised routine perinatal care combined with the web-based “Mamma Mia” program.	significant improvement in depressive symptoms was observed.
Qin et al. (61)	CareMom application	28 “daily challenges”, with users completing one challenge per day.	4 weeks	(1) psychoeducation; (2) cognitive restructuring.	Significant improvement in depressive symptoms was observed.
Kim et al. (62)	Avecmom mobile application	daily sessions of 10–15 minutes.	4 weeks	(1) breathing meditation; (2) body scan; (3) emotion-awareness meditation; (4) loving-kindness meditation.	Significant improvement in depressive symptoms was observed.

“-” indicates not reported.

## 4 Application of ICBT for negative emotions in pregnant women

### 4.1 Anxiety

Anxiety is one of the most prevalent negative emotions during pregnancy. If left untreated in a timely manner, it can exert adverse effects on both maternal and neonatal health, and increase the risk of preterm birth, low birth weight, and postpartum depression (63). Accordingly, effective alleviation of antenatal anxiety is of great significance for protecting maternal and child health. The UK National Institute for Health and Care Excellence (64) and the National Collaborating Centre for Mental Health (65) recommend CBT as the first-line clinical intervention for antenatal anxiety. Although traditional in-person face-to-face CBT has been verified to be effective in reducing anxiety symptoms among pregnant individuals, accumulating emerging evidence supports the therapeutic effect of ICBT for this population (53). A Japanese pilot randomized controlled trial focusing on a midwife-led online CBT program reported statistically significant improvements in anxiety symptoms among the intervention group of pregnant women (44). Shariatpanahi et al. (8) implemented a randomized controlled trial (RCT), in which 82 pregnant women with anxiety were randomly assigned to two groups: the standalone internet-based emotion-focused CBT (iECBT) group, and the iECBT combined with spousal participation group (where husbands received 8 weekly 20-minute intervention sessions via an independent online link). The results indicated that iECBT effectively reduced anxiety symptoms regardless of whether spousal participation was included, which demonstrates that ICBT exerts a reliable therapeutic effect on antenatal anxiety and does not require a high level of family support as a prerequisite, suggesting that this intervention has broad application prospects. A meta-analysis conducted by Lau et al. (54) further confirmed that therapist-supported ICBT can effectively reduce stress, anxiety, and depressive symptoms among perinatal women. However, existing research on ICBT intervention for antenatal anxiety is still relatively insufficient. Most current studies are constrained by small sample sizes, and generally lack long-term follow-up outcome data. Future research is required to implement large-sample, multi-center RCTs, and to further explore the combined intervention effect of ICBT and other adjuvant strategies including mindfulness intervention and group support.

### 4.2 Depression

Depressive symptoms represent a prevalent condition among pregnant populations. Although the majority of existing studies (40, 61, 66) corroborate the beneficial effect of ICBT in the management of antenatal depression, considerable heterogeneity is observed across reported outcomes, necessitating in-depth stratified analysis. Kim et al. (62) reported that the Avecmom application, which contains four mindfulness training modules to be completed over a four-week period, significantly reduced depressive symptom severity and anxiety levels among pregnant individuals with depression, indicating that “lightweight” ICBT interventions are clinically feasible for this population. Takae et al. (40) reached consistent positive conclusions utilizing the Luna Luna Baby application, despite the restriction that the ICBT program was only accessible after 16 weeks of gestation, with one 5-minute module provided per week. Similarly, Mahoney et al. (67) evaluated the THIS WAY UP ICBT program, which consists of only three online sessions, among 1502 perinatal women, and demonstrated that ICBT yielded significant improvements in perinatal depressive symptoms, further supporting the clinical potential of brief ICBT interventions. This result is consistent with the findings of Kim et al. (62) obtained via the Avecmom application, which consolidates the evidence for the feasibility of “lightweight” ICBT in pregnant populations. However, PandaMom, the first internet- and mobile-based guided intervention platform for perinatal depression in Germany, has thus far only been validated for feasibility, and its intervention efficacy still requires verification through large-scale randomized controlled trials (RCTs) (42), a conclusion that is consistent with the findings reported by Nishi et al. (41). Heterogeneities in intervention content and sample characteristics may account for these inconsistent findings across studies. In conclusion, the efficacy of ICBT for antenatal depression may be modulated by multiple factors, including intervention intensity, content, outcome measurement instruments, and sample characteristics. Future research is required to compare different intervention protocols and identify subgroups of pregnant women that are most likely to respond to ICBT, so as to achieve precision intervention.

### 4.3 Fear of childbirth

Fear of childbirth (FOC) is a common negative emotional experience among pregnant populations, which can occur across the antenatal, intrapartum, and postpartum periods. This condition negatively impacts maternal physical and psychological health and may also induce adverse outcomes for the fetus, highlighting the necessity of timely intervention. Existing studies indicate that ICBT has a significant effect on alleviating FOC in pregnant women (24, 68). For example, Abdelaziz et al. (2) investigated a structured online childbirth education course (completion within 6 weeks, covering 6 modules including audio resources, video demonstrations, teaching videos, researcher guidance and homework assignments) among pregnant women in Egypt. The results showed that the intervention significantly reduced FOC levels in the third trimester, which is consistent with the research conclusions reported by Shahsavan et al. (46). This effect may be attributed to the accessibility and efficiency of online CBT, which enables pregnant women to identify and adjust negative cognitions related to childbirth via cognitive restructuring. Accordingly, providing accessible online intervention programs in the third trimester is of great importance for supporting pregnant women to approach childbirth with positive cognition.

In addition, Ashton et al. (12) delivered a brief online group-based Acceptance and Commitment Therapy (ACT) intervention for primiparous women with FOC, which consisted of two sessions held on Sunday evenings via the Zoom platform. However, a meta-analysis conducted by Neo et al. (69) reached inconsistent conclusions: at 6–8 weeks postpartum, no statistically significant differences in stress and FOC levels were observed between the internet-based psychological intervention group (including cognitive behavioral therapy, metacognitive therapy, positive psychology, etc.) and the control group. This inconsistency may result from the fact that the meta-analysis only included 4 to 6 randomized controlled trials involving 404 to 531 pregnant women, and the relatively limited sample size may have reduced the statistical power of the analysis. Although previous systematic reviews (70) have confirmed that in-person educational interventions can effectively reduce FOC, research on internet-based psychological interventions for this population is still insufficient. The aforementioned inconsistent findings can provide a reference for the development and implementation of internet-based psychological interventions in future research. Overall, the effectiveness of ICBT for FOC still requires further verification through high-quality studies with large sample sizes.

## 5 Conclusion

Internet-based cognitive behavioral therapy (ICBT), which incorporates the core principles of cognitive behavioral therapy (CBT) into internet-based technological frameworks, shows considerable application potential in alleviating negative emotional symptoms including anxiety, depression and FOC among pregnant women. Existing research evidence suggests that, compared with traditional face-to-face CBT interventions, ICBT can break through the constraints of geographical space and time, reduce intervention implementation costs, and partially alleviate the contradiction between supply and demand of public mental health resources, which renders it particularly suitable for perinatal mental health services.

Nevertheless, the application of ICBT in the field of pregnant women’s mental health still faces multiple unsolved problems. First, there is a lack of unified standards for intervention frequency, duration and content, which reduces the comparability of research results and limits the extrapolation of research conclusions. Second, the absence of trimester-specific stratified intervention protocols remains a notable gap; the optimal timing for initiating ICBT and the differential prioritization of intervention content across gestational stages warrant further investigation. Third, the sample size of most existing studies is generally small, and large-sample, multi-center randomized controlled trials (RCTs) are still insufficient. Fourth, relevant research in China is still in the exploratory initial stage, and the localization adaptation and practical promotion of intervention programs that conform to Chinese cultural characteristics are still insufficient.

Future research needs to further clarify the optimal intervention model of ICBT, systematically explore its applicability for pregnant women in different gestational trimesters, and integrate emerging technologies such as artificial intelligence and machine learning to realize the personalized customization of interventions. At the same time, it is necessary to comprehensively evaluate intervention compliance, long-term intervention effects and maternal and infant outcomes, so as to promote the systematic integration of ICBT into clinical practice of perinatal mental health, and provide more scientific, accessible and effective intervention strategies for maternal mental health.
